# Unisexual Reproduction of *Cryptococcus gattii*

**DOI:** 10.1371/journal.pone.0111089

**Published:** 2014-10-22

**Authors:** Sujal S. Phadke, Marianna Feretzaki, Shelly Applen Clancey, Olaf Mueller, Joseph Heitman

**Affiliations:** Department of Molecular Genetics and Microbiology, Duke University, Durham, North Carolina, United States of America; Woosuk University, Republic of Korea

## Abstract

*Cryptococcus gattii* is a basidiomycetous human fungal pathogen that typically causes infection in tropical and subtropical regions and is responsible for an ongoing outbreak in immunocompetent individuals on Vancouver Island and in the Pacific Northwest of the US. Pathogenesis of this species may be linked to its sexual cycle that generates infectious propagules called basidiospores. A marked predominance of only one mating type (α) in clinical and environmental isolates suggests that **a**-α opposite-sex reproduction may be infrequent or geographically restricted, raising the possibility of an alternative unisexual cycle involving cells of only α mating type, as discovered previously in the related pathogenic species *Cryptococcus neoformans*. Here we report observation of hallmark features of unisexual reproduction in a clinical isolate of *C. gattii* (isolate 97/433) and describe genetic and environmental factors conducive to this sexual cycle. Our results are consistent with population genetic evidence of recombination in the largely unisexual populations of *C. gattii* and provide a useful genetic model for understanding how novel modes of sexual reproduction may contribute to evolution and virulence in this species.

## Introduction

Cryptococcosis is a pathological condition involving pneumonia and meningitis caused by the basidiomycetous fungi *Cryptococcus neoformans* and *Cryptococcus gattii* [1], [2]. While immunocompromised patients are at risk for infection by *C*. *neoformans*, some infections do occur in immunocompetent hosts [3], [4], [5], [6]. *C. gattii* is frequently associated with infection of immunocompetent hosts making it a primary pathogen but some molecular types (VGIII, VGIV) may be more commonly associated with HIV/AIDS patients [7], [8], [9], [10], [11]. Recently, the VGII molecular type is responsible for the outbreak that began on Vancouver Island and has now spread to the Pacific Northwest of the United States [12], [13], [14], [15], [16]. Disease is initiated upon inhalation of infectious propagules in the form of desiccated yeast cells or basidiospores, which are produced during sexual reproduction; hence, understanding the genetic and environmental factors conducive to the sexual cycle is critical to exploring its contributions to the pathogenicity of these fungi [17], [18].

A traditional sexual cycle between the opposite **a** and α mating types has been described in both *C. neoformans* and *C. gattii* [19], [20], [21], [22]. However, like *C. neoformans*, most clinical and environmental isolates of *C. gattii* are of the α mating type and fewer are of the **a** mating type, indicating 1) infrequent opportunity for opposite-sex mating and 2) an association of the α mating type with virulence [11], [23], [24], [25]. Population genetic analyses show evidence of recombination and sexual reproduction in largely unisexual populations of *C. gattii* [11], [15], [26], [27], which led us to hypothesize that, like *C. neoformans*, strains of the α mating type of *C. gattii* may have the ability to produce infectious spore particles via unisexual reproduction [28], [29], [30].

*C. neoformans* and *C. gattii* usually proliferate as haploid budding yeasts during vegetative growth and can also form pseudohyphae (elongated yeast cells with characteristic mother-daughter cell attachments) in response to nutrient-limitation or the presence of predatory amoebae [31], [32]. True septate hyphae with clamp cells are produced during opposite-sex mating triggered by various environmental cues including the presence of pheromones, the sugar inositol, copper, darkness, and pigeon guano [33], [34]. A third hyphal phenotype known as haploid filamentation or monokaryotic fruiting was discovered in strains of the α mating type of *C. neoformans* induced by similar environmental cues but was initially thought to be asexual [35]. Later studies demonstrated a central role of meiosis during monokaryotic fruiting verifying its sexual nature and this developmental pathway has been termed α-α unisexual reproduction [30], [36]. Although induced by similar environmental cues as opposite-sex mating, unisexual reproduction in *C. neoformans* is characterized by distinctive morphological hallmarks including formation of monokaryotic hyphae with unfused clamp connections in contrast to dikaryotic hyphae and fused clamp cells produced during opposite-sex mating [29]. Also, similar genetic circuits, including the MAPK pathway activated by pheromone-receptor interactions and the cAMP pathway induced by nutrient-limitation, play pivotal roles in both unisexual and opposite-sex reproduction [37].

Unisexual reproduction has been discovered in two fungal pathogens: *C. neoformans* [30] and *Candida albicans* [38]. In *C. neoformans*, this novel cycle can begin with diploidization of cells of the α mating type via α-α cell fusion or endoreplication followed by meiosis, and shares similarities with the traditional opposite-sex mating in inducing morphogenic changes involving the yeast to hyphal transition and basidiospore formation. Thus, the unisexual cycle may contribute to virulence of *C. neoformans* through generation of infectious spores and adaptive genetic variation [21], [28], [29]. Population genetic evidence supports occurrence of unisexual mating in natural populations of *C. neoformans* [39], [40]. Importantly, sexual reproduction has been implicated in origins of *C. gattii* hypervirulent genotypes, necessitating investigation of the sexual cycles in the various molecular subgroups of this species [41], [42], [43], [44], which are distinct enough to represent four or five different taxa.

Among the four molecular types (VGI-VGIV) of *C. gattii*, the abundance of the α mating type is especially striking in the VGIIIa subgroup, which is prevalent in Mexico and California and has only one isolate of the **a** mating type reported to date, making it a candidate subgroup to include isolates that may undergo unisexual reproduction [11], [45], [46]. This speculation is further supported by the evidence of recombination suggesting instances of sexual reproduction within the VGIIIa population in the near absence of the opposite mating type. VGIII isolates are also fertile in **a**-α mating and typically more so than other VG types [11], [20]. The remaining subgroups including VGI, VGII, VGIIIb, and VGIV represent genetically (and in some cases geographically) subdivided populations but show a similar skew towards the α mating type, presenting distinct opportunities for the occurrence of unisexual reproduction. Here, we report the discovery of morphological hallmarks of unisexual reproduction in a self-fertile, VGIIIa clinical isolate of *C. gattii* (97/433), and provide genetic evidence and define environmental factors suggesting occurrence of the unisexual cycle in this pathogen.

## Materials and Methods

### Strains and culture conditions

The strains used in our analysis are listed in Table 1. Self-fertility was tested by making a patch of the respective strain with a sterile toothpick on fresh Murashige Skoog (MS) medium (Sigma CATALOG #M5524) followed by incubation in the dark at room temperature for 3 weeks to 2 months unless stated otherwise. Plates were incubated facing up and without parafilm to avoid accumulation of condensation and CO_2_ that can inhibit sexual reproduction. Fertility was assessed by light microscopic examination, specifically for the formation of hyphae, blastospores, basidia, and basidiospores, at the periphery of the patch.

**Table 1:** Strains utilized in this study.

Strain	Genetic background	Genotype	Sources/Reference
R265	*C. gattii* VGIIa	*MAT*α	[13]
YL4	*C. gattii* VGIIa	*MAT*a	[42]
NIH312	*C. gattii* VGIIIb	*MAT*α	[20]
97/433	*C. gattii* VGIIIa	*MAT*α	[70]
B4546	*C. gattii* VGIIIb	*MAT*a	[20]
SP118	97/433/YL4	*MAT*α/*MAT*a	This study
H99	*C. neoformans* var. *grubii*	*MAT*α	[75]
JEC21	*C. neoformans* var. *neoformans*	*MAT*α	[76]
SP95	97/433	*MAT*α *gpa3*Δ::*NAT* #*1*	This study
SP99	97/433	*MAT*α *gpa3*Δ::*NAT* #*2*	This study
SP104	97/433	*MAT*α *crg1*Δ::*NAT* #*1*	This study
SP107	97/433	*MAT*α *crg1*Δ::*NAT* #*2*	This study
SP134	97/433	*MAT*α P*_GPD1_*-*ZNF2 NEO AMP*	This study
SP135	97/433	*MAT*α P*_GPD1_*-*MAT2 NEO AMP*	This study
SP136	97/433	*MAT*α P*_GPD1_*-*MAT2 NEO AMP*	This study
SP137	97/433	*MAT*α P*_GPD1_*-*CPR2 NEO AMP*	This study
RDC1	JEC21	*MAT*α *cpk1*::*ADE2 lys2 ade2*	[52]
RDC5	JEC21	*MAT*α *cpk1*::*ADE2 ade2*	[52]
XL926	JEC21	*MAT*α *mat2*Δ::*NAT*	[57]
XL576	JEC21	*MAT*α *znf2*Δ::*NAT*	[57]
XL577	JEC21	*MAT*α *znf2*Δ::*NAT*	[57]

Wildtype and mutant strains generated during the course of this study are listed with specific mutations that were introduced.

### Microscopy

Hyphae were fixed using 3.7% formaldehyde and PBS with 1% Triton, stained with DAPI (Sigma), and imaged with a Zeiss Axio Imager A1 fluorescence microscope equipped with an AxioCam MRM Digital camera. Scanning electron microscopy (SEM) samples were processed as follows: 1mm^3^ blocks of edges of the colonies were excised, washed with 0.1 M Na cacodylate buffer (pH = 6.8) and incubated in fixation buffer at 4°C followed by rinsing in cold 0.1 M Na cacodylate buffer three times, post-fixation in 2% osmium tetroxide–0.1 M Na cacodylate buffer for 2.5 hr at 4°C, critical point drying, and sputter coating before being viewed by SEM.

### Gene deletion

Overlap PCR products were generated by combining the nourseothrecin (NAT) drug resistance marker amplified from plasmid pAI3 and 5′ and 3′ flanking sequences of the genes of interest (*GPA3* or *CRG1*) from strain 97/433. Wildtype or derived mutant strains were biolistically transformed with the overlap PCR products (Table 1). Transformants in which the gene of interest had been replaced by homologous recombination with the overlap PCR product were identified using ORF amplification (absent in deletion strains) and 5′ or 3′ junction specific PCR analysis (present in deletion strains) (Figure S2).

### Gene overexpression

Strain 97/433 was biolistically transformed with the plasmids pDX64, pDX59, or pYPP19 to introduce the *C. neoformans* alleles of the *MAT2, ZNF2*, or *CPR2* genes respectively, under the control of the *C. neoformans* constitutive promoter *GPD1* along with the NEO drug resistance marker. We verified stable integration by testing NEO resistance of ∼100 clones per transformant following rounds of non-selective passage on YPD agar (100/100 NEO resistant  = 100% stability during mitotic non-selective passage) and verified overexpression of the genes using qPCR with primers specific to the introduced *C. neoformans* alleles.

### Ploidy determination

We used Flourescent Activated Cell Sorting (FACS) to assess ploidy. Cells grown in YPD medium were washed with 1X PBS buffer, and then fixed in 1 ml of 70% ethanol overnight at 4°C. Cells were then washed with 1 ml of NS buffer (10 mM Tris-HCl (pH = 7.5), 250 mM sucrose, 1 mM EDTA (pH = 8.0), 1 mM MgCl_2_, 0.1 mM CaCl_2_, 0.1 mM ZnCl_2_) and stained overnight with propidium iodide (10 mg/ml) in 1X NS buffer containing RNaseA (1 mg/ml) at room temperature. 0.5 ml of stained cells were diluted into 0.5 ml of 50 mM Tris-HCl (pH = 8.0). Flow cytometry analysis was performed using the FL1 channel with a Becton-Dickinson FACScan (Duke University Medical Center Flow Cytometry Core Facility). Ploidy was further investigated *in silico* by copy number variation analysis as described [43]. Reads were mapped against WM276, with 97/433 as query, and NIH312 as reference.

### Genome analysis

16,416,655 100 bp Illumina paired reads were quality filtered with ea-utils (Aronesty, 2011, http://code.google.com/p/ea-utils). Filtered reads were assembled with Velvet v. 1.2.10 [47] and SOAPdenovo2 [48] respectively. The resulting draft assembly had 734 scaffolds (>100 bp) with an N50 of 200.2 kb. Scaffolds were mapped to the publicly available *MAT*α locus of *C. gattii* NIH312 [11] with MUMmer v. 3.23 [49]. The resulting 115,760 bp supercontig contained the 97/433 *MAT*α locus and the two flanking genes *FAO1* and 02431. Synteny between the *MAT*α loci of NIH312, 97/433, and WM276 was evaluated by pairwise alignment in MUMmer. The sequence reads have been deposited at the NIH sequence read archive (SRA) with Genbank Accession number SRR1392241.

### qPCR analysis

RNA was isolated from 5 ml overnight cultures incubated for 48 hours at room temperature in liquid YPD or MS agar medium in the dark. Cells were harvested and lyophilized overnight prior to extraction using TRIzol Reagent following the manufacturer's instructions (Invitrogen). 2 µg of total RNA was treated with Turbo DNAse (Ambion), and single-stranded cDNA was synthesized using AffinityScript RT-RNAse (Stratagene). Quantitative Real-Time PCR (RT-PCR) assays were performed in triplicate on an Applied Biosystems 7500 Real-Time PCR System using Brilliant SYBR Green qRT-PCR master mix (Stratagene). A no template control and melting curves were analyzed to exclude primer artifacts. Expression of the pheromone gene was normalized relative to that of the endogenous control, *GPD1*, and the level of expression was determined using the 2^−ΔΔCT^ approach. The primers used for RT-PCR are listed in Table S2. The Student's t-test was used to establish significance of differences in expression of the pheromone in the mutants compared to the wild type (significance p<0.05).

### Heat-induced filamentation

Strains were grown in YPD liquid cultures at 30°C for 24 hrs, washed, and spotted on solid YPD media. The cultures were incubated at 30°C, 37°C, and 38°C for 2, 3, 4, and 5 days in the dark. Cells from each condition were transferred on MS, Filamentation agar, and 5% V8 juice agar medium (pH = 7) and incubated for 5, 10, 15, and 20 days in the dark at room temperature. The hyphae were visualized with a Nikon Eclipse E400 and photographed using a Nikon Digital Camera DXM1200F.

## Results

### Identification of *C. gattii* strain capable of monokaryotic fruiting

Our aim was to screen for the ability to undergo unisexual reproduction in a broad collection of *C. gattii* isolates and define environmental conditions supporting this developmental pathway. We surveyed a total of 128 strains from the four *C. gattii* VGI-VGIV molecular types for a self-fertility phenotype under various environmental conditions known to support unisexual and heterosexual reproduction of *C. neoformans* (Table S1). In some cases, VGII isolates were incubated adjacent to an opposite mating type strain (YL4 *MAT* **a**) as a source of Mf**a** pheromone to activate the pheromone-signaling cascade and stimulate hyphal development. In other cases, VGIII isolates were tested in confrontation with a self-fertile α/**a** diploid or an αX**a** mating mixture in close proximity as a source of both pheromones.

One clinical isolate, 97/433 belonging to the subgroup VGIIIa of *C. gattii* was observed to produce hyphae in solo-culture on mating inducing MS and V8 pH = 5.0 media, and the production of hyphae was accelerated by confrontation with strains producing the **a** pheromone alone or both the α and **a** pheromones. 97/433 is a clinical strain of the α mating type of *C. gattii* isolated from a 17 year old HIV/AIDS female patient in Mexico (provided by Dr. Francoise Dromer, Pasteur Institute). Multilocus sequence typing analysis places 97/433 in the VGIIIa subgroup [11] and this isolate was confirmed to be haploid based on FACS analysis (Figure S1A). The other isolates did not produce hyphae in solo-culture under the conditions tested.

### Genome analysis of *C. gattii* strain, 97/433

**Figure 1:**
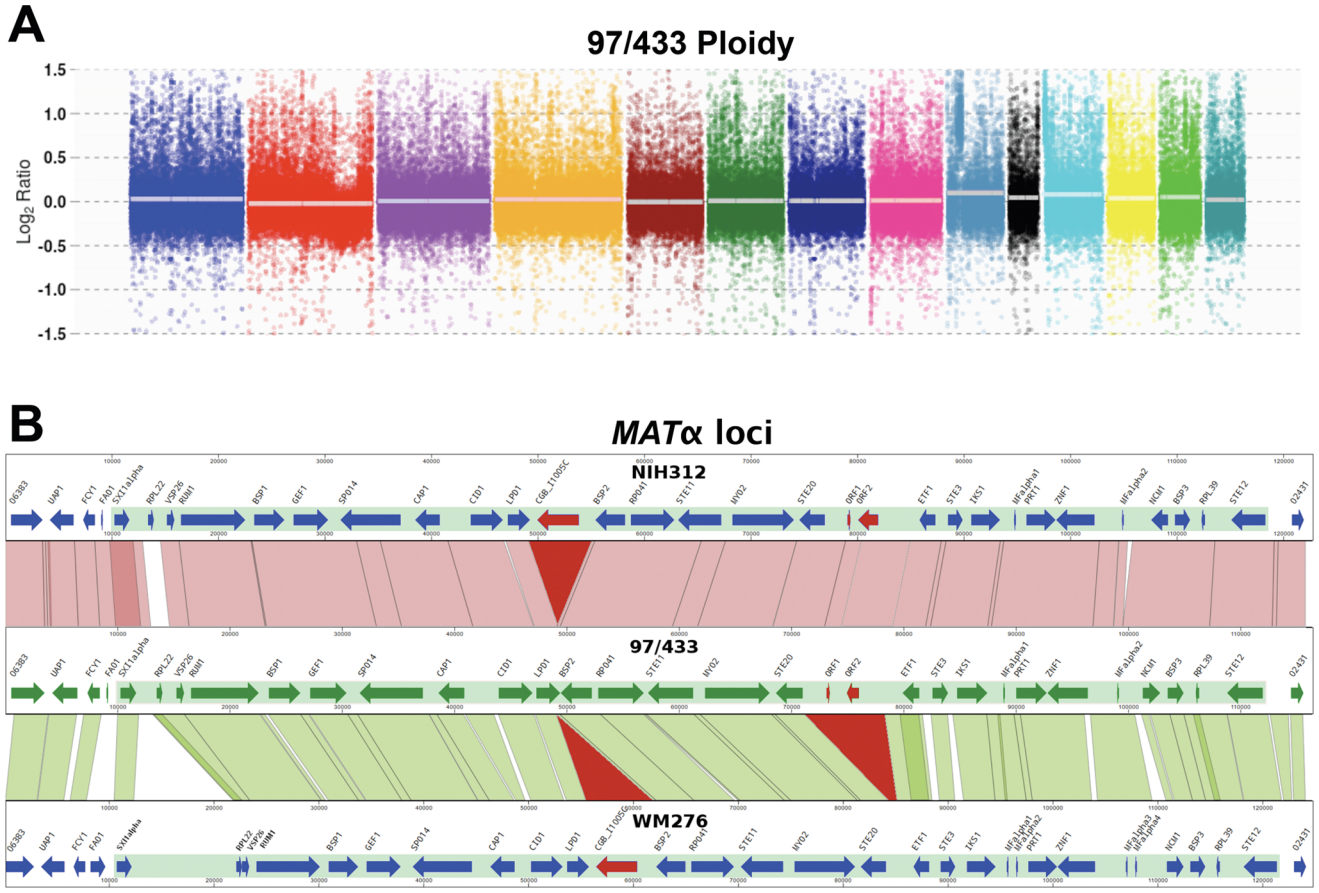
Genome analysis of 97/433. (**A**) Copy number variation analysis of next generation sequencing reads confirms that 97/433 is a haploid *MAT*α strain. Each of the 14 chromosomes is represented as a different color in a rainbow pattern. The average of the read coverage is a white horizontal bar. A log2 ratio close to zero for each chromosome compared to the genome average combined with FACS data support assignment as a haploid, euploid strain. (**B**) *MAT*α comparison between 97/433 (VGIIIa), NIH312 (VGIIIb), and WM276 (VGI) revealed a 6 kb deletion in 97/433 and a larger intergenic region between the *STE20* and *ETF1* genes.

We investigated the genome of 97/433 for aneuploidy and for the presence of alleles found in the opposite mating type (**a**) as possible causes of monokaryotic fruiting/self-fertility. We performed high-throughput Illumina sequencing to analyze the genome of 97/433 and compared it with other sequenced strains from *C. gattii* and *C. neoformans* [44], [45], [50]. Genome analysis confirmed that 97/433 is a *C. gattii* VGIIIa strain of mating type α. Copy number variation analysis (Figure 1A) shows that 97/433 is a euploid haploid isolate, eliminating duplicated aneuploid regions as a possible factor contributing to monokaryotic fruiting/self-fertility. Furthermore, using sequences of JEC21α/JEC20**a** (*C. neoformans* var. *neoformans*), NIH312α, and B4546**a** in BLAST searches, we found that 97/433 lacks mating type **a**-specific genes, including *NCP1* **a**, the pheromone encoding gene *MF* **a**, and the *SXI2* **a** gene, which encodes a homeodomain protein that controls cell identity and sexual development in *Cryptococcus*. The *MAT* locus of 97/433 contains only mating type α-specific genes including the pheromone gene *MF*α and the mating-type specific MAP kinase pathway genes of the pheromone-signaling cascade (*STE20*α, *STE11*α, and *STE12*α) (Figure 1B).

To compare the genomic organization of the *MAT*α locus we conducted a synteny analysis of the *MAT*α loci of 97/433 (VGIIIa), NIH312 (VGIIIb), and WM276 (VGI). Although, we observed full synteny between all selected *MAT*α strains, regarding gene content and orientation, there were two major differences between the strains. There was an approximately 6 kb deletion in the 97/433 *MAT*α locus, between the *LPD1* and *BSP2* genes with respect to WM276 and NIH312. This region (Figure 1B, red triangle) encodes a hypothetical protein in WM276 and NIH312 (CGB_I1005C, Genbank acc. XP_003196318 [50]), which is missing from 97/433 and has not been previously described for the *MAT*α locus. Moreover, the intergenic regions between the *STE20* and *ETF1* genes are considerably larger in NIH312 and 97/433 (∼9 kb) than in WM276 (∼2.7 kb), and contain two hypothetical genes (*ORF1*, *ORF2*), which are absent in WM276. *ORF1* is a short, single exon gene, encoding an 84 amino acid peptide of unknown function. *ORF2* contains a glycosyl transferase domain (Pfam acc. Pfam13632). The protein is conserved in other basidiomycete fungi but contain N- and C-terminal truncations (97/433: 284 aa, NIH312: 504 aa) compared to the most similar sequence found in *Tremella mesenterica* DSM_1558 (814 aa).

### Detailed characterization of strain 97/433 reveals hallmark morphological features of unisexual reproduction

**Figure 2:**
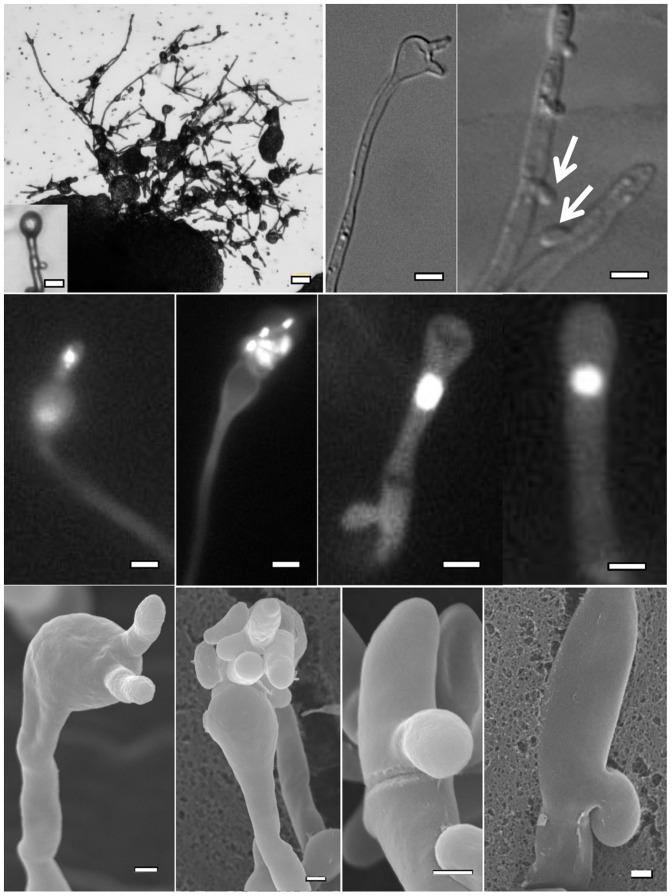
Hallmarks of unisexual reproduction in VGIIIa mating type α strain 97/433. From left to right, (Top panel) Peripheral hyphae and basidium (inset), basidiospores, unfused clamp cells (arrow); (Middle panel) DAPI stained basidiospores, monokaryotic hyphae; (Bottom panel) SEM of basidiospores, unfused clamps; Scale bar = 1 µm; except for top left image (10 µm).

We tested various combinations of media, temperatures, and light conditions, including V8 pH = 5.0 agar, Murashige Skoog (MS) medium, and filament agar and found 97/433 to produce hyphae on V8 pH 5.0 and MS media at room temperature (24°C) in the dark. When solo cultured under these conditions for up to ∼2 months, 97/433 develops hyphae at isolated spots along the periphery of the yeast colony (Figure 2). Although, the hyphal formation phenotype was highly reproducible, not every colony of 97/433 tested produced hyphae or to the same extent.

By providing a source of **a** or α+**a** pheromones nearby (**a** cells or an α X **a** mating mixture of 97/433 with strain B4546 or YL4), hyphal development was accelerated with hyphae beginning to emerge 10 days earlier than when strains were plated with no pheromone source. Similar observations have been made for unisexual reproduction in *C. neoformans* [30], [51], [52], suggesting the pheromone-response pathway is involved in monokaryotic fruiting/self-fertility of 97/433.

The DNA content of the vegetative yeast cells (blastospores) emerging from the hyphal cells reveal the ploidy of the hyphae. Multiple blastospores were isolated from 10 different hyphae along with 10 basidiospores and subjected to FACS analysis. We found that 97/433 generates haploid hyphae with a single nucleus that yielded haploid blastospores and basidiospores (Figure S1B and Figure 2). These findings suggest that the nucleus in the haploid hyphae diploidizes in the basidia prior to meiosis to generate four haploid progeny (Figure 2).

Based on both light and scanning electron microscopic examinations, we observed hallmarks of unisexual reproduction including monokaryotic hyphae and unfused clamp cells along with the presence of blastospores, basidia, and occasional basidiospores (Figure 2). As observed in *C. neoformans* [30], sporulation during unisexual reproduction of the *C. gattii* VGIII isolate 97/433 was found to be inefficient compared to bisexual reproduction, and was found to only contain isolated individual or collapsed basidiospore chains (Figure 2). Along with aerial hyphae, 97/433 occasionally produced invasive hyphae during solo-culture, a phenotype distinct from unisexually reproducing strains of *C. neoformans*.

### Signaling pathways involved in self-fertility of 97/433

We hypothesized that the pheromone activated MAP kinase pathway would play an active role in unisexual reproduction of 97/433. The MAPK pathway is a conserved signaling cascade that orchestrates mating in both ascomycetes and basidiomycetes [52]. This pathway is instrumental in the progression of *Cryptococcus* through the sexual cycle and the components of the MAPK pathway signal coordinately with the G-protein regulated cAMP pathway to regulate opposite-sex and unisexual reproduction.

**Figure 3:**
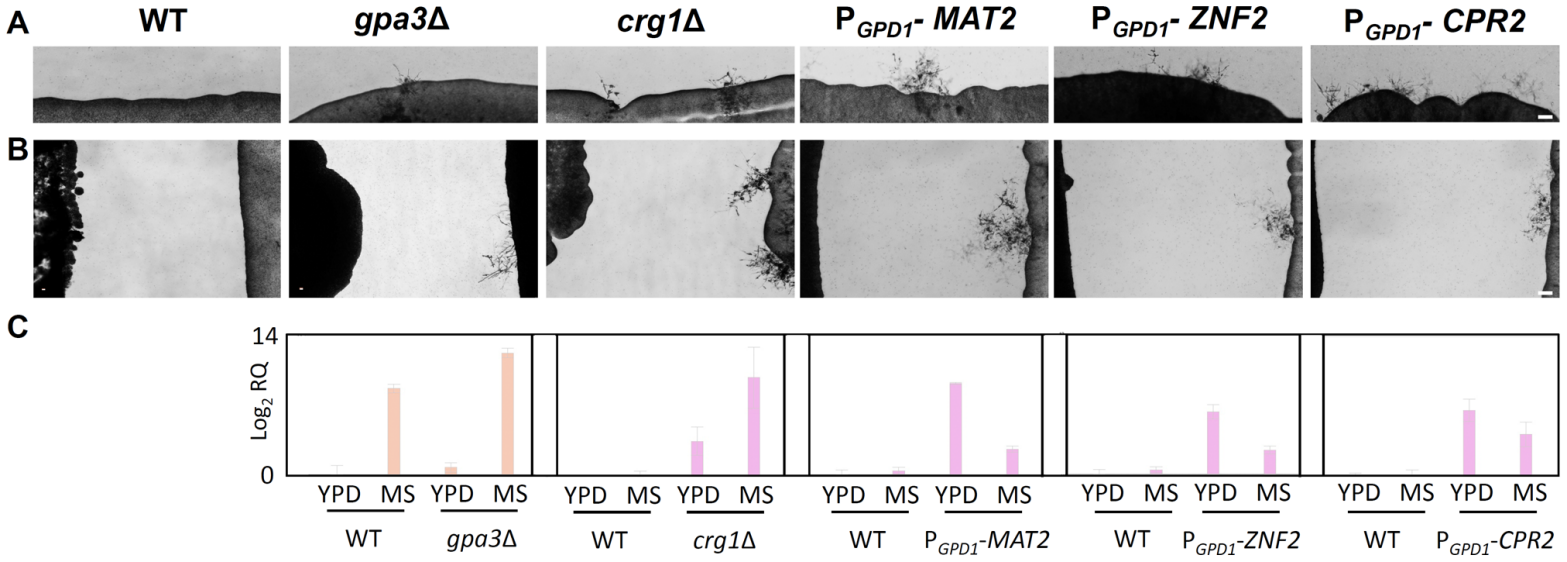
MAPK pathway is involved in unisexual reproduction of 97/433. *gpa3*Δ and *crg1*Δ mutations, and overexpression of *MAT2, ZNF2*, or *CPR2* all enhanced self-fertility of 97/433. (**A**) Two independent *gpa3*Δ and *crg1*Δ mutants were analyzed (see also Figure S2). Solo-cultures or (**B**) confrontation assays with a partner of the **a** mating type (YL4**a** on the left side of each panel) were incubated on MS agar for 3 weeks at room temperature (24°C) in the dark and photographed. Scale bar = 50 µm. (**C**) Mutants were analyzed for elevated expression of the *MF*α pheromone gene as compared to the wildtype after 48 hours incubation on MS medium (C).

We targeted two negative regulators of the MAPK pathway including the *CRG1* gene, which encodes a regulator of G-protein signaling (RGS) that attenuates the pheromone response [20], [53] and *GPA3*, which encodes one of the three Gα subunits that interact to regulate Cpk1-dependent hyphal formation during mating in *C. neoformans* [54], [55], [56]. Deletion of either gene resulted in significantly higher pheromone expression than wild-type and led to enhanced and accelerated hyphal growth in solo-culture and confrontation assays of these 97/433 mutants on MS agar (21 days for *gpa3*Δ or *crg1*Δ vs ∼60 days for wild-type in solo-cultures) (Figure 3A and 3B, Figure S1). These observations show that activation of the pheromone response pathway enhances monokaryotic fruiting of *C*. *gattii* (Figure 3C), consistent with unisexual reproduction as the underlying mechanism.

We also targeted three positive regulators of the α unisexual and **a**-α bisexual reproduction cascades including the transcription factors Mat2 and Znf2, which act in the mating pathway downstream of Cpk1 [57], and Cpr2, which is a homologue of the pheromone receptor Ste3 that can activate both sexual cycles in a ligand-independent fashion [58]. When the *C. neoformans* alleles of *MAT2*, *ZNF2*, or *CPR2* under the control of the *GPD1* promoter was introduced into strain 97/433 by biolistic transformation, the formation of hyphae and spores was accelerated in solo culture on MS medium in as few as 18 days (compared to ∼60 days for wild-type) and this was associated with increased pheromone expression (Figure 3A and 3B, Figure S2). These results show that the intrinsic ability of 97/433 to produce hyphae in solo-cultures is enhanced upon transgene or mutational activation of the mating pathway and further supports the conclusion that 97/433 undergoes unisexual reproduction.

### Growth at high temperature induces hyphal development in 97/433

Previous studies have shown that a dimorphic transition of *C. neoformans* can be stimulated by growth at high temperature through an unknown pathway that is independent of sexual reproduction. Hyphal development is induced by high temperature (37–40°C) and is associated with G2 cell cycle arrest [59], [60]. In a report by Fu et al., the authors found that only G2-arrested cells (isolated by growth at high temperature or in the presence of the G2 cell cycle arrest agent nocodazole) generated haploid hyphae with few basidiospores [60].

**Figure 4:**
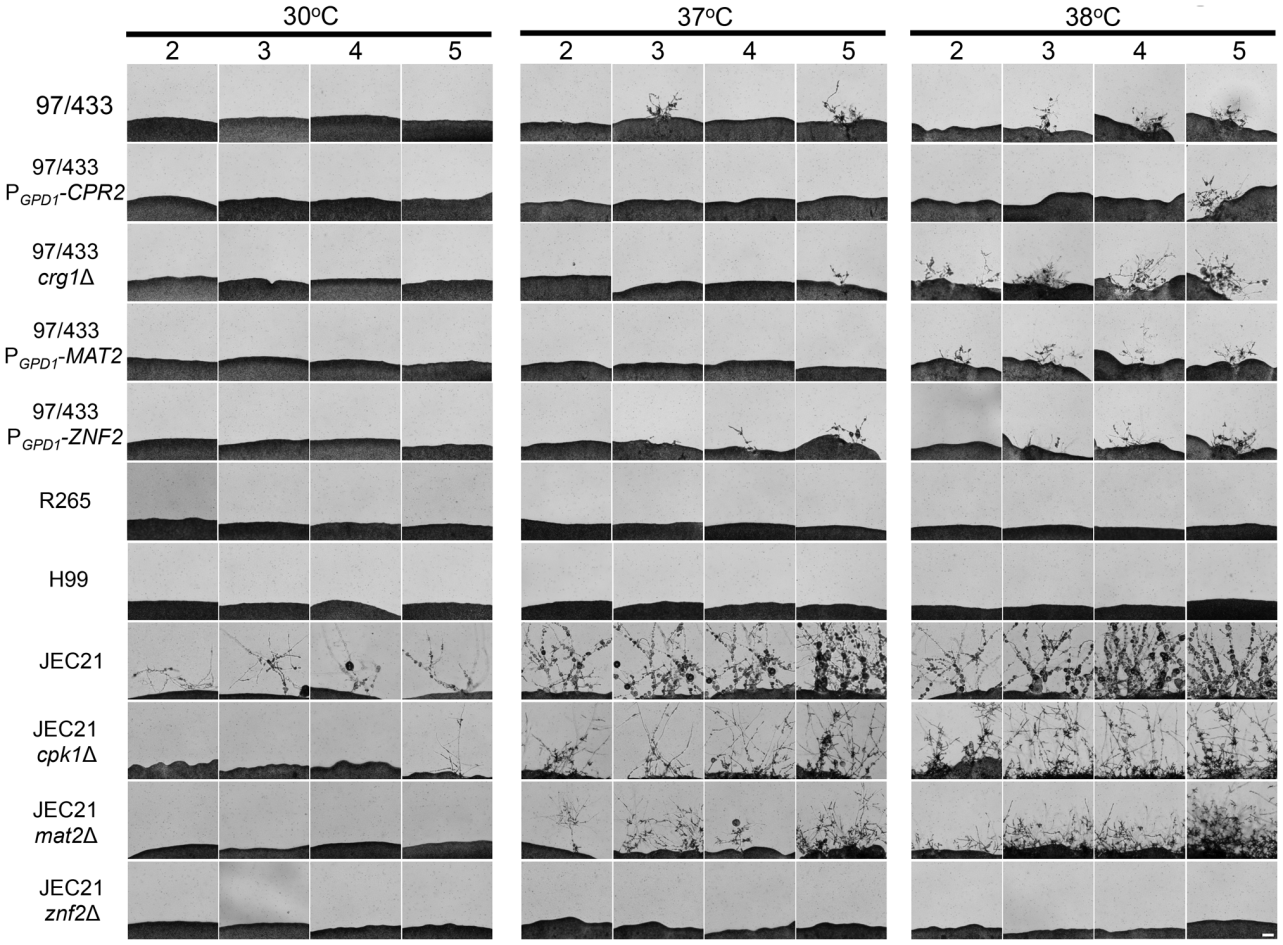
Growth at high temperature stimulates hyphal development. The cells were grown on solid YPD media at 30°C, 37°C and 38°C for 2, 3, 4, and 5 days in the dark. Solo cultures were spotted on MS media and incubated at room temperature for 5 days in the dark and hyphal development was visualized by microscopy and photographed. Scale bar = 50 µm.

To investigate the role of high temperature in hyphal development we incubated *C. gattii* VGII R265, VGIII 97/433, *C. neoformans* var. *grubii* H99, and *C. neoformans* var. *neoformans* JEC21 strains on rich YPD media at 30°C, 37°C, and 38°C for 2, 3, 4, and 5 days in the dark. The seed cultures were spotted on MS, Filamentation agar, or V8 media pH = 7.0 and incubated in the dark, at room temperature. We found that high temperature stimulates filamentation in 97/433 with hyphae appearing as soon as 5 days post inoculation of solo cultures (Figure 4). Deletion of *CRG1* or overexpression of *MAT2* or *ZNF2* in 97/433 increased hyphal development generating more condensed and longer hyphae at the periphery of the culture. On the other hand, neither the *C. neoformans* var. *grubii* strain H99 nor the *C. gattii* strain R265 strains generated heat-induced hyphae following prolonged incubation in different nutrient limiting media (Figure 4).

JEC21 belongs to the non-pathogenic self-fertile group *C. neoformans* var. *neoformans*. Unisexual reproduction has been observed in var. *neoformans* strains under laboratory conditions. Higher temperature enhanced hyphal development in JEC21 generating hyphae following prolonged incubation at 30°C. Deletion of major regulators of the pheromone pathway, *CPK1* or *MAT2*, in JEC21 reduced but did not abolish filamentation, indicating that the components of the pheromone-signaling cascade contribute to heat-induced morphogenesis but are not strictly required (Figure 4). Interestingly, deletion of *ZNF2*, which governs morphogenesis during sexual development, abolished heat-induced hyphal development. These results indicate that prior growth at high temperature may enhance filamentation in nutrient limiting conditions via pathways distinct from those that govern unisexual reproduction.

Heat-induced morphogenesis was observed in strains known to undergo unisexual reproduction. During unisexual reproduction the cells fuse or undergo endoreplication, generating a diploid intermediate product that ultimately undergoes meiosis followed by multiple rounds of mitosis and budding to produce the recombinant progeny, the basidiospores. Meiosis and sporulation are hallmarks of sexual development and they are strictly regulated by genetic elements [30], [36]. To determine whether heat-induced hyphal development leads to production of spores we incubated the heat-induced solo cultures on nutrient limiting media for ∼2 weeks. Visualization of the hyphae under the microscope at high magnification revealed that the majority of hyphae produced no basidia (Figure 4). A very few basidia were observed at the apex of aerial hyphae but were characterized by the absence of spores or spore chains (Figure S4). Prolonged incubation on different nutrient-limiting media did not yield any basidiospores in any of the strains tested. In addition, heat-induced filamentation in JEC21 resulted in hyphae covered with yeast cells that appear to originate from germination of blastospores that emerged from the hyphae (Figure 3S). Based on these results we conclude that growth at high temperature induces both invasive and aerial hyphae. However, the aerial hyphae fail to produce basidiospores or chains of spores on the surface of basidia, possibly due to an absence of meiosis and suggesting that this heat-induced morphological pathway may be an asexual process.

## Discussion

Sexual reproduction is a principle strategy adopted by pathogens to generate an assortment of virulent progeny [61]. It provides an indirect long-term advantage by generating genetically diverse progeny adapted to different hosts [62], [63], [64]. Sexual reproduction may also contribute to virulence of human fungal pathogens, including *C. neoformans* and *C. gattii*, by generating, for example, infectious propagules in the form of spores. Adaptations that facilitate sexual reproduction are commonly observed in fungi and include various forms of homothallism such as mating type switching, mating type locus fusion, and the presence of both mating type loci in a single genome. Hallmarks of unisexual reproduction in *C. gattii* illustrate another example of this phenomenon and indicates a central role for this sexual cycle in pathogenesis of this species.

In our screen, only 1 out of 128 *C. gattii* isolates was found to be capable of monokaryotic fruiting reminiscent of unisexual reproduction. This low proportion is consistent with the overall lower fertility observed in *C. gattii* in comparison with *C. neoformans*. For example, in a study of 120 clinical and environmental *C. gattii* isolates, the majority was found to be infertile, even during opposite-sex mating [22]. Also, our observation may be analogous to the finding of only a low frequency (∼3%) of isolates capable of undergoing white to opaque switching in *C. albicans* [38], [65], which we now appreciate are the rare minority **a**/**a** and α/α MTL homozygous isolates compared to the majority of **a**/α MTL heterozygous isolates that do not undergo white to opaque switching. Hence, although unisexual reproduction could be infrequent or limited to specific genotypes in *C. gattii*, different strains may undergo unisexual reproduction under specific environmental conditions not explored in our studies. Furthermore, the absence of aneuploidy or any **a** mating type specific genes in the genome is consistent with models in which selfing in 97/433 may be a quantitative trait and contingent upon the overall genetic composition such that many genes, each with a small effect may interact to contribute to this ability. In this context, 97/433 provides a useful genetic tool for studying unisexual reproduction in *C. gattii*. Similar cases in the past have included the discovery of pseudohyphal development in the Σ1278b strain of *Saccharomyces cerevisiae*. While many lab adapted strains are incapable of developing this phenotype, the wild isolate Σ1278b has been an important research model [66], [67]. In another example, the sister species *C. neoformans MAT* **a** strain 125.91 was found to mate with only 3 out of 150 *MAT*α isolates tested under laboratory conditions, leading to the discovery of the sexual cycle in *C. neoformans* var. *grubii* and generation of congenic strains [68]. Thus, there is a history of studies on seemingly anomalous strains that ultimately leads to insights into the life cycles and sexual cycles of fungi. Similarly, we propose 97/433 as a foundation for further studies on *C. gattii* unisexual reproduction.

*C. gattii* contains four (VGI-VGIV) molecular groups and 97/433 belongs to the subgroup VGIIIa. Although this subgroup was thought more likely to contain strains capable of unisexual reproduction, partly due to the near absence of the opposite **a** mating type [11], strong population genetic evidence for unisexual reproduction is available for additional *C. gattii* lineages [27]. The geographic ranges of VGII and VGIII rarely overlap indicating that opportunities for interbreeding may be limited [69]. Opposite-sex mating between VGII and VGIII has been observed in the laboratory but results in low germination and viability of the spore progeny. Also, the absence of hybrids in previous studies has suggested that sexual reproduction in *C. gattii* tends to occur within rather than between the VG types [41]. In this context, our study presents a direct demonstration of the unisexual cycle in a specific molecular group of *C. gattii*.

Previous studies and our analysis of the mating type (*MAT*) locus in 97/433 indicate a unique *SXI1*α allele similar to other VGIII isolates. The majority of VGIII strains harbor a mutation in the *SXI1*α gene that introduces a premature stop codon in the C-terminus domain that truncates the protein [70]. Surprisingly, the truncated version of the *SXI1*α allele is functional in the VGIII group, which contains most of the fertile strains of *C. gattii* species [11], [70], indicating that this allele of the homeodomain protein may enhance fertility. Further comparison of the 97/433 (VGIIIa), NIH312 (VGIIIb), and WM276 (VGI) genomes revealed two interesting variations in the *MAT*α loci. There is an approximately 6 kb deletion in the 97/433 *MAT*α locus located between the *LPD1* and *BSP2* genes. The respective region in NIH312 and WM276 encodes an unknown hypothetical protein with no conserved domains. BLAST analysis of the predicted open reading frame sequence showed low homology with proteins involved in oxidative phosphorylation and amino acid metabolism and it does not appear likely to play an obvious role in sexual development. The intergenic region between the *STE20* and *ETF1* genes is significantly larger in 97/433 and NIH312. Interestingly this region contains two *in silico* predicted, unknown peptides (Orf1 and Orf2) that have not been previously described in the *MAT*α locus and Orf2 contains a known conserved glycosyl transferase domain.

The previous finding that unisexual reproduction in the sister species *C. neoformans* is a form of sexual cycle has relied on two lines of genetic evidence involving demonstration of 1) diploidization via α-α cell fusion or endoreplication and 2) meiosis [30]. Our failure to detect diploid fusion products using various mutants of 97/433 suggests that diploidization in *C. gattii* may mainly occur via endoreplication or karyogamy in the basidia. This speculation is further supported by the lack of diploid blastospores but the presence of haploid basidiospores indicating that monokaryotic hyphae in 97/433 likely contain a haploid nucleus, which we hypothesize diploidizes in the basidium prior to meiosis (Figure S1). Also consistent with this conclusion is the near absence of α/α diploids in natural populations of *C. gattii*, with the exception of strain RB59 which appears homozygous throughout the genome [41], [44]. An alternative possibility is the absence of a meiotic cycle and in this case hyphal development in 97/433 may represent a haploid monokaryotic phenotype. Further studies, including evidence for involvement of meiotic machinery such as Spo11, are required to verify that monokaryotic fruiting of strain 97/433 indeed represents unisexual reproduction.

Hyphal development has been directly associated with sexual development. In the absence of an opposite mating partner hyphal development was initially thought to be asexual [35]; however, diploidization through cell-cell fusion or endoreplication is associated with hyphal development, meiosis, and sporulation to generate recombinant or aneuploid progeny [30], [36]. Unisexual reproduction has been observed previously in strains of *C. neoformans* var. *neoformans* under laboratory conditions. Interestingly, recent studies showed that growth at high temperature or in the presence of nocodazole, which both cause a G2 cell cycle arrest, may induce hyphal development in a mating-independent manner [59], [60]. Growth of 97/433 and its derived mutants at 37°C on rich media exhibited enhanced hyphal development in solo cultures on nutrient limiting media at room temperature. However, these heat-induced hyphae failed to generate spores or chains of spores in any of the strains tested, including the JEC21 strain that belongs to the self-fertile *C. neoformans* var. *neoformans* group. Heat induction generated both invasive and aerial hyphae. Invasive hyphae are differentiated structures that support the growth of aerial hyphae, allow foraging for nutrients in the substrate, and they are not physically associated with the fruiting bodies. On the other hand, the absence of basidiospores on the apex of aerial basidia may reflect an asexual mechanism that appears to be common in strains that retain the ability to undergo a dimorphic transition during unisexual reproduction.

Involvement of the pheromone response pathway as observed in our studies is reminiscent of similar observations in the sister species *C. neoformans* [52], [57] and in the ascomycetous fungal pathogen *C. albicans* [38]. Our candidate gene approach reveals that downstream components of this pathway including Mat2 and Znf2 as well as the constitutively active GPCR, Cpr2 are conserved between *C. neoformans* and *C. gattii* and can accelerate unisexual reproduction across the species boundary when heterologously expressed. Future studies will reveal whether these results can be extended to include the third pathogenic specie of the complex *C. neoformans* var. *grubii*.

Consistent with previous observations in *C. neoformans* and *C. gattii* VGII isolates [20], [71], *CRG1* acts as a negative regulator of the pheromone response pathway in VGIII. Crg1 is a homolog of the *S. cerevisiae* GTPase activating protein Sst2 and attenuates pheromone response by stimulating GTPase activity of the Gα subunits Gpa2 and Gpa3 during mating. Thus, deletion of the *CRG1* gene prolongs the half-life of active Gα-GTP subunit complex and enhances the pheromone response facilitating unisexual reproduction of 97/433 [53]. Similarly, we found that deleting the gene encoding the Gα subunit Gpa3 also enhanced hyphal growth in 97/433. Previous studies have found a role for Gpa3 in the pheromone response MAPK pathway and deletion of the *GPA3* gene enhances mating of *C. neoformans* [54], [55]. This reveals that the signaling pathways involved in these sexual cycles act as plastic developmental networks that could be further modified during adaptation of pathogens to specific environmental cues.

Our results indicate that unisexual reproduction involves signal transduction pathways and facilitates dimorphic transitions and formation of invasive hyphae in *C. gattii*. Earlier studies have hypothesized the possibility of selfing in clonal populations of another fungal pathogen *Pneumocytis carinii* and discovered selfing in microbial parasites including *Leshmania*, *Trypanosoma*, and *Plasmodium* [72], [73], [74]. Taken together with the discovery of unisexual reproduction in *C. neoformans* and *C. albicans*, our findings highlight transitions in modes of sexual reproduction in an increasing number of fungal pathogens suggesting that more such examples in fungal and other microbial eukaryotic pathogens likely remain to be discovered.

## Supporting Information

Figure S1 **Ploidy of 97/433 yeast cells, blastospores, and basidiospores.** (**A**) Flow cytometry reveals that 97/433 is a haploid strain during vegetative growth. (**B**) The vegetative cells (blastospores) emerging from the hyphae reflect the DNA content of the hyphae. FACS analysis of multiple blastospores showed that the nuclei of 97/433 hyphae are haploid in solo cultures. FACS analysis of germinated basidiospores revealed they are haploid. JEC21 (1n, haploid control); XL143 (2n, diploid control); Nuclear DNA content is indicated by 1n (haploid) and 2n (diploid). The *x*-axis indicates fluorescence intensity reflecting DNA content, and the *y*-axis indicates cell counts.
(TIF)

Figure S2 **Verification of mutant genotypes.** (**A**) *gpa3*Δ and *crg1*Δ deletions were generated by overlap PCR and introduced by biolistic transformation and homologous recombination in the VGIII mating type α strain 97/433. (**B**) (Top panel) Deletion mutants yielded a specific product in 5′ Junction PCR; (middle panel) deletion mutants lacked the ORF and failed to yield a PCR product with primers within the ORF; (Bottom panel) *SXI1*α served as a PCR control. (**C**) Relative levels of *MAT2* and *ZNF2* expression in strains transformed with the respective genes expressed under the control of the *GPD1* promoter. Strains overexpressing *MAT2* and *ZNF2* show elevated expression levels of the respective genes as compared to the wildtype in both YPD and MS media.
(TIF)

Figure S3 **Enhanced self-fertility of independent** ***gpa3*** **Δ and** ***crg1*** **Δ mutants.** Independent mutants were analyzed to establish mutant phenotypes are attributable to the introduced mutation. Solo-cultures (Top panel) or confrontation assays (Bottom panel) were incubated on MS agar for 20 days at room temperature in the dark and photographed at 4X magnification. Scale bar = 50 µm.
(TIF)

Figure S4 **Sporulation is blocked in heat-induced hyphae.** Heat-induced solo-cultures were incubated on MS agar for 20 days at room temperature in the dark. The hyphae and basidia were visualized by light microscopy and photographed at 20X magnification. Scale bar = 10 µm.
(TIF)

Table S1 **List of strains tested for self-fertility in three media including V8, Filament agar (FA) and Murashige Skoog medium (MS).** All tests were conducted in dark. Self-fertility was tested in the presence of light for some isolates as indicated. + signifies that self-fertility was tested under the respective environmental condition. – represents that the respective strain was not tested under the given conditions.
(XLSX)

Table S2 **Primers used in this study for overlap PCR, qPCR, and verification of gene deletion.**
(DOC)
