# Supplementary figures and images for: Unisexual Reproduction of Cryptococcus gattii
Source: PLoS One. 2014 Oct 22;9(10):e111089. doi: 10.1371/journal.pone.0111089 (PMC4206507; doi:10.1371/journal.pone.0111089)

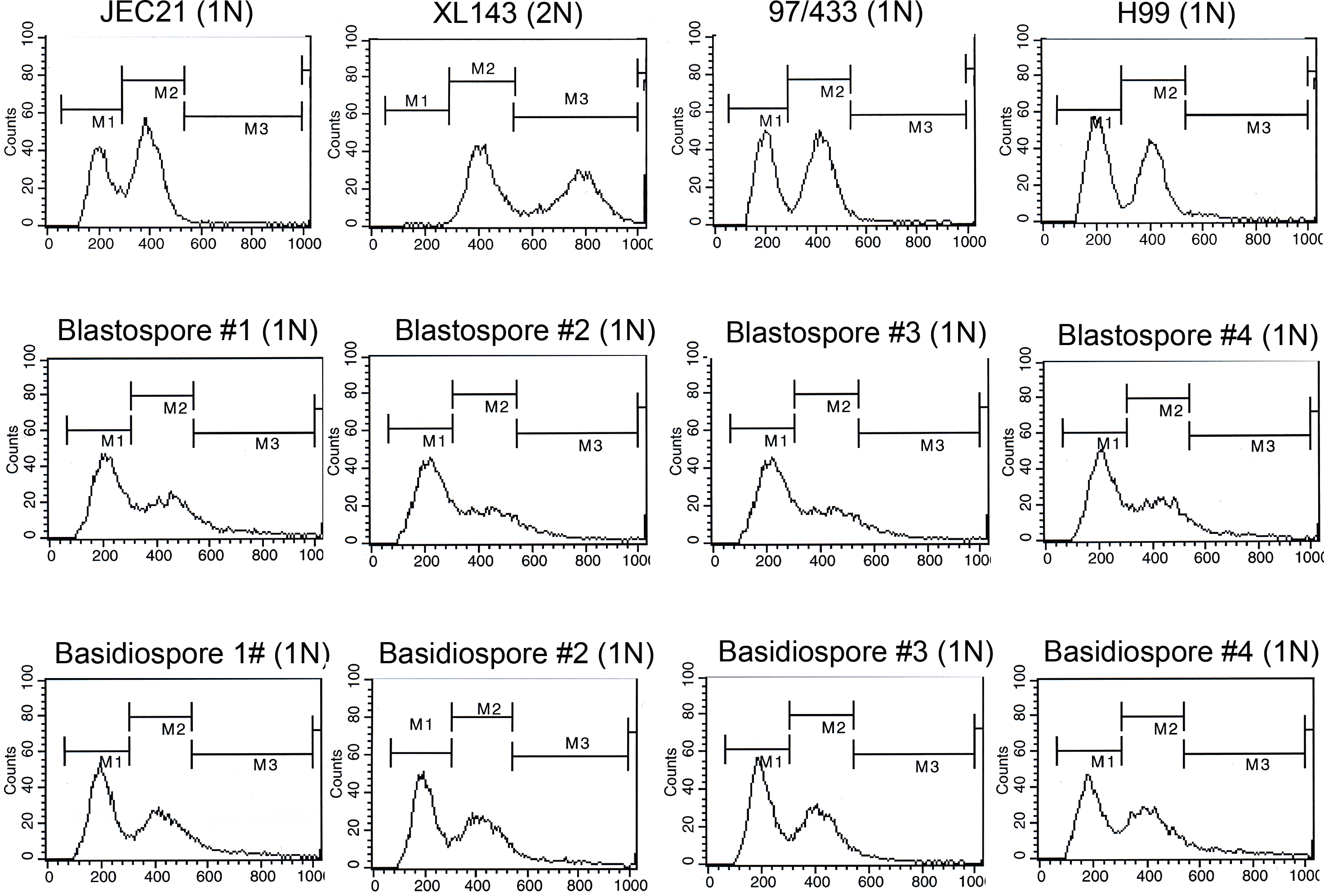

Supplement: Figure S1 — Ploidy of 97/433 yeast cells, blastospores, and basidiospores. (A) Flow cytometry reveals that 97/433 is a haploid strain during vegetative growth. (B) The vegetative cells (blastospores) emerging from the hyphae reflect the DNA content of the hyphae. FACS analysis of multiple blastospores showed that the nuclei of 97/433 hyphae are haploid in solo cultures. FACS analysis of germinated basidiospores revealed they are haploid. JEC21 (1n, haploid control); XL143 (2n, diploid control); Nuclear DNA content is indicated by 1n (haploid) and 2n (diploid). The x-axis indicates fluorescence intensity reflecting DNA content, and the y-axis indicates cell counts. (TIF) [file pone.0111089.s001.tif]

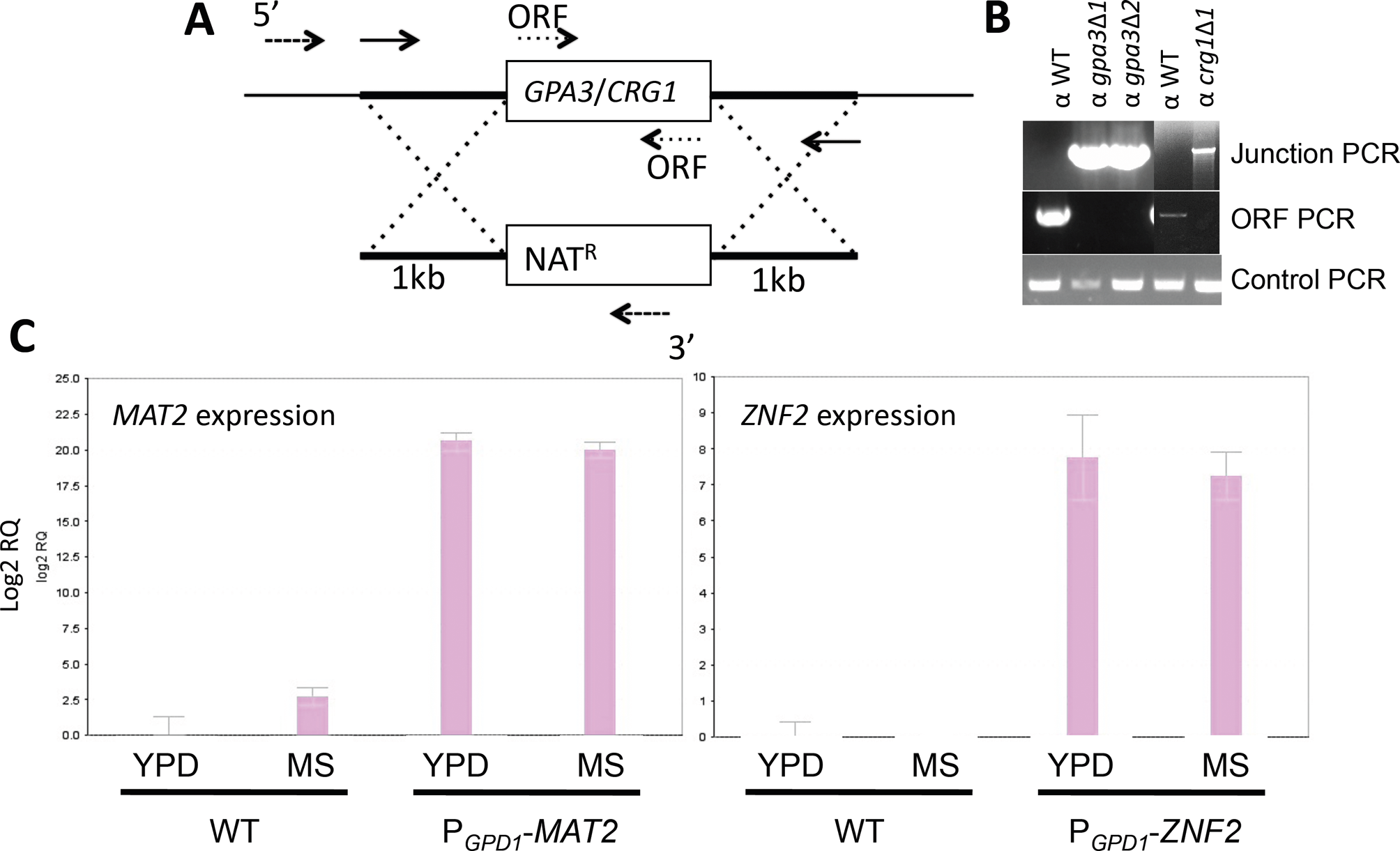

Supplement: Figure S2 — Verification of mutant genotypes. (A) gpa3Δ and crg1Δ deletions were generated by overlap PCR and introduced by biolistic transformation and homologous recombination in the VGIII mating type α strain 97/433. (B) (Top panel) Deletion mutants yielded a specific product in 5′ Junction PCR; (middle panel) deletion mutants lacked the ORF and failed to yield a PCR product with primers within the ORF; (Bottom panel) SXI1α served as a PCR control. (C) Relative levels of MAT2 and ZNF2 expression in strains transformed with the respective genes expressed under the control of the GPD1 promoter. Strains overexpressing MAT2 and ZNF2 show elevated expression levels of the respective genes as compared to the wildtype in both YPD and MS media. (TIF) [file pone.0111089.s002.tif]

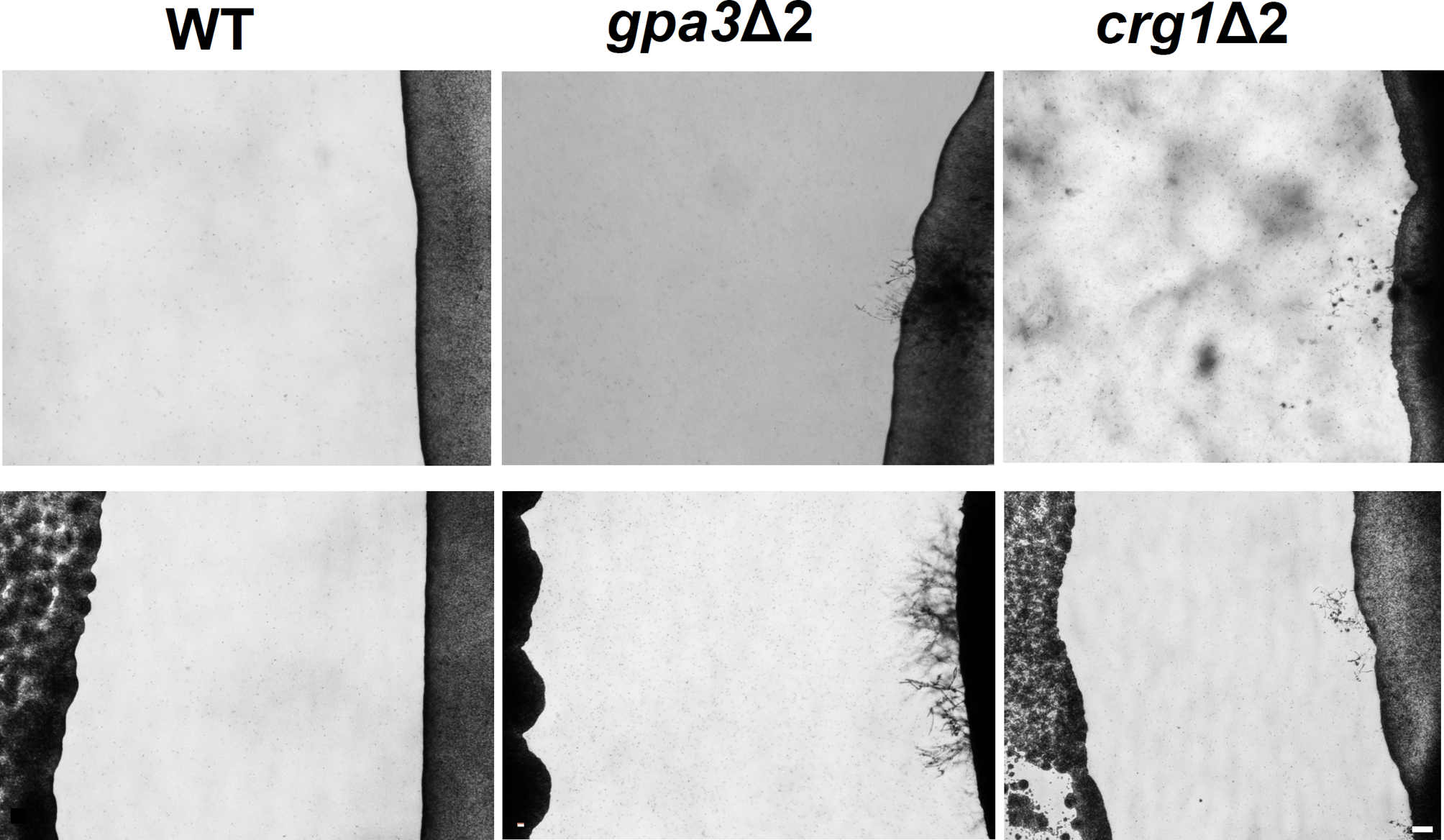

Supplement: Figure S3 — Enhanced self-fertility of independent gpa3 Δ and crg1 Δ mutants. Independent mutants were analyzed to establish mutant phenotypes are attributable to the introduced mutation. Solo-cultures (Top panel) or confrontation assays (Bottom panel) were incubated on MS agar for 20 days at room temperature in the dark and photographed at 4X magnification. Scale bar = 50 µm. (TIF) [file pone.0111089.s003.tif]

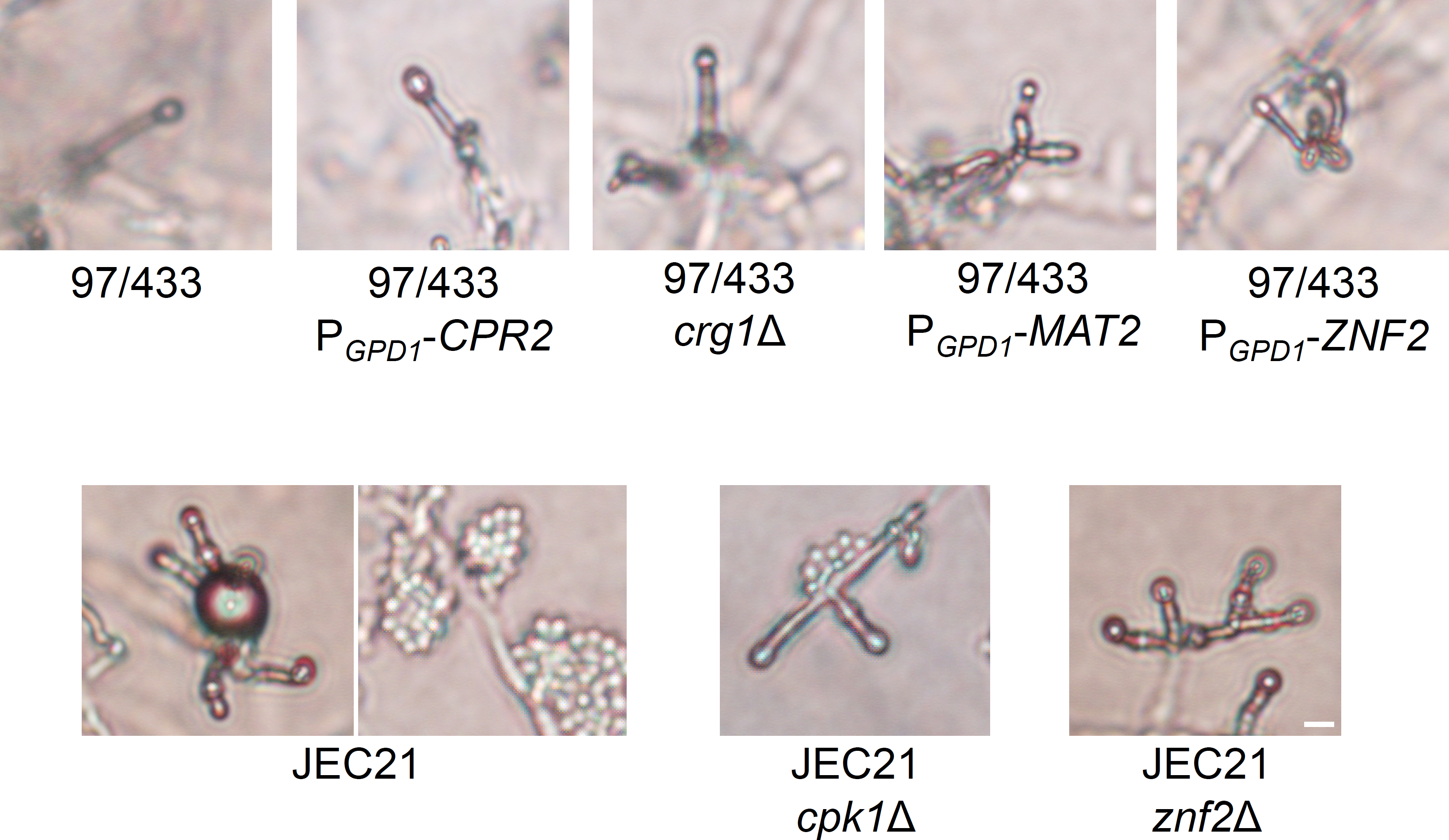

Supplement: Figure S4 — Sporulation is blocked in heat-induced hyphae. Heat-induced solo-cultures were incubated on MS agar for 20 days at room temperature in the dark. The hyphae and basidia were visualized by light microscopy and photographed at 20X magnification. Scale bar = 10 µm. (TIF) [file pone.0111089.s004.tif]
