# Supplementary material for: Unisexual Reproduction of Cryptococcus gattii
Source: PLoS One. 2014 Oct 22;9(10):e111089. doi: 10.1371/journal.pone.0111089 (PMC4206507; doi:10.1371/journal.pone.0111089)
Supplement: Table S2 — Primers used in this study for overlap PCR, qPCR, and verification of gene deletion. (DOC) [file pone.0111089.s006.doc]

**Table S2.** Primers used in this study

| **Primer name** | **Sequence (5’ to 3’)** | **Description** |
| --- | --- | --- |
| M13F | GTAAAACGACGGCCAG |  |
| M13R | CAGGAAACAGCTATGAC |  |
| JOHE38683 | CAGTGAATGCTGATCACTGGCTTC | *GPA3* disruption |
| JOHE38729 | CTGGCCGTCGTTTTACCAGAGTTGATCGTTGCTGGTATGCGCA | *GPA3* disruption |
| JOHE38720 | CTGGCCGTCGTTTTACCAGGTAATGTATACCAAGTCGGGA | *GPA3* disruption |
| JOHE38695 | GTCTCCTTCTCCTCCTCTTCCT | *GPA3* disruption |
| JOHE38680 | ACGGTACTAGTCAAGTTGTCTATAGATGCGTC | *GPA3* screening |
| JOHE38681 | TCGTCTATATTTGTTGATCCCT | *GPA3* screening |
| JOHE38663 | ACGGAGCAACAACAAATGGGTATCGAC | *CRG1* disruption |
| JOHE38720 | CTGGCCGTCGTTTTACCAGGTAATGTATACCAAGTCGGGA | *CRG1* disruption |
| JOHE38721 | CTGGCCGTCGTTTTACGAGTGAGGAGAATGGAAGGGT | *CRG1* disruption |
| JOHE38661 | ACGGGATTCAGGCACGATTTCAGGCACT | *CRG1* disruption |
| JOHE38662 | AGTCCGCACCACTACAACCAG | *CRG1* screening |
| JOHE21081 | CTGGTTCTGTAACGGTAAGCC | *CRG1* screening |
| JOHE38893 | TCGGCCGTGTACCAAACTTAACCAAG | *CRG1* screening |
| JOHE38894 | TGCGAGATAGTGAGGTATGCGCGAGTAGGACT | *CRG1* screening |
| JOHE23667 | GTCAACCTCGATGCCTACAAACCG | *GPD1* RT-PCR |
| JOHE23668 | GCCTACAGCCTTAGCAGCACCA | *GPD1* RT-PCR |
| JOHE24434 | CTACCGACCAGCAACCAACCATCGCTAC | *MF*α*1* RT-PCR |
| JOHE24435 | GTCAATACCATCTAAACAAGTCCCATACGCTTC | *MF*α*1* RT-PCR |
| JOHE39703 | TCGCTCGACGCAACACCTTGCTC | *ZNF2* RT-PCR |
| JOHE39704 | CTGGGACAACATAGACGCAGTG | *ZNF2* RT-PCR |
| JOHE39705 | TGCTCTTCCAACTGCCCATC | *CPR2* RT-PCR |
| JOHE39706 | ACGGGAGCGCCGATGGTGACTG | *CPR2* RT-PCR |
| JOHE38698 | ATGGATACCGAGATCGAGG | *MAT2* RT-PCR |
| JOHE38699 | TCAAGACCGACCATTGAAACAC | *MAT2* RT-PCR |
